# Evaluation of peri-plaque pericoronary adipose tissue attenuation in coronary atherosclerosis using a dual-layer spectral detector CT

**DOI:** 10.3389/fmed.2024.1357981

**Published:** 2024-03-11

**Authors:** Yulin Jia, Lei Zou, Ming Xue, Xiaoyu Zhang, Xigang Xiao

**Affiliations:** ^1^Department of Radiology, The First Affiliated Hospital of Harbin Medical University, Harbin, China; ^2^Department of Radiology, Zigong Fourth People's Hospital, Zigong, China

**Keywords:** pericoronary adipose tissue, fat attenuation index, coronary inflammation, longitudinal location, dual-layer spectral detector CT

## Abstract

**Purpose:**

This study aimed to evaluate the differences between pericoronary adipose tissue (PCAT) attenuation at different measured locations in evaluating coronary atherosclerosis using spectral computed tomography (CT) and to explore valuable imaging indicators.

**Methods:**

A total of 330 patients with suspicious coronary atherosclerosis were enrolled and underwent coronary CT angiography with dual-layer spectral detector CT (SDCT). Proximal and peri-plaque fat attenuation index (FAI) of stenosis coronary arteries were measured using both conventional images (CIs) and virtual monoenergetic images (VMIs) ranging from 40 keV to 100 keV. The slopes of the spectral attenuation curve (λ) of proximal and peri-plaque PCAT at three different monoenergetic intervals were calculated. Additionally, peri-plaque FAI on CI and virtual non-contrast images, and effective atomic number were measured manually.

**Results:**

A total of 231 coronary arteries with plaques and lumen stenosis were finally enrolled. Peri-plaque FAI_CI_ and FAI_VMI_ were significantly higher in severe stenosis than in mild and moderate stenosis (*p* < 0.05), while peri-plaque λ, proximal FAI, and proximal λ were not statistically different. Proximal FAI, peri-plaque FAI, and peri-plaque λ were significantly higher in low-density non-calcified plaque (LD-NCP) and non-calcified plaque (NCP) than in calcified plaque (*p* < 0.01). Peri-plaque FAI was the highest in the LD-NCP group, while proximal FAI was the highest in the NCP group. In severe stenosis and in LD-NCP, peri-plaque FAI was significantly higher than proximal FAI (*p* < 0.05). The manually measured parameters related to peri-plaque PCAT attenuation had a positive correlation with the results of peri-plaque FAI measured automatically.

**Conclusion:**

Peri-plaque PCAT has more value in assessing coronary atherosclerosis than proximal PCAT. Peri-plaque PCAT attenuation is expected to be used as a standard biomarker for evaluating plaque vulnerability and hemodynamic characteristics.

## Introduction

According to the 2021 World Health Organization report, cardiovascular disease (CVD) is the world’s leading cause of death, with 85% of deaths resulting from heart attacks and strokes (1). As the most common CVD, coronary atherosclerotic heart disease (CAD) is receiving increasing attention, especially in the aspect of accurate diagnosis and treatment. Traditionally, the assessment of CAD using coronary computed tomography angiography (CCTA) focuses mainly on the morphological evaluation of plaque types and lumen stenosis. Recent studies have shown that CAD is a chronic inflammatory disease that can release mediators into pericoronary adipose tissue (PCAT), causing morphological changes and increased attenuation of PCAT (2, 3). PCAT is visceral adipose tissue, which contacts the coronary artery directly. PCAT was defined as an epicardial adipose tissue (EAT) with a radial distance of 1 mm from the outer wall of the coronary artery, and all voxel values were within the range of −190 to −30 HU (4). PCAT has a complex bidirectional paracrine pathway with the coronary artery wall. The dysfunctional PCAT, which reflects the inflammatory status of the PCAT and the coronary artery, can also be detected by CCTA according to the increased PCAT attenuation (4, 5). Thus, some studies measured the perivascular fat attenuation index (FAI) of coronary arteries to assess the plaque risk or severity of CAD (6–9). Most of them adopted FAI around the proximal right coronary artery (RCA) due to the abundance of adipose tissue, which can be obtained and measured easily compared to the left anterior descending (LAD) artery and left circumflex (LCX) artery. Other studies used FAI of proximal segments of three major coronary arteries or around the plaque that caused the maximal degree of vascular stenosis as the evaluation indicator (10–12). At present, there is no unified standard in terms of measurement locations of PCAT attenuation for the evaluation of CAD-related ischemic severity or risk. Therefore, the aim of this study is to evaluate the diagnostic value of PCAT attenuation at different positions based on spectral CT for CAD.

## Materials and methods

### Patients

**Figure 1:**
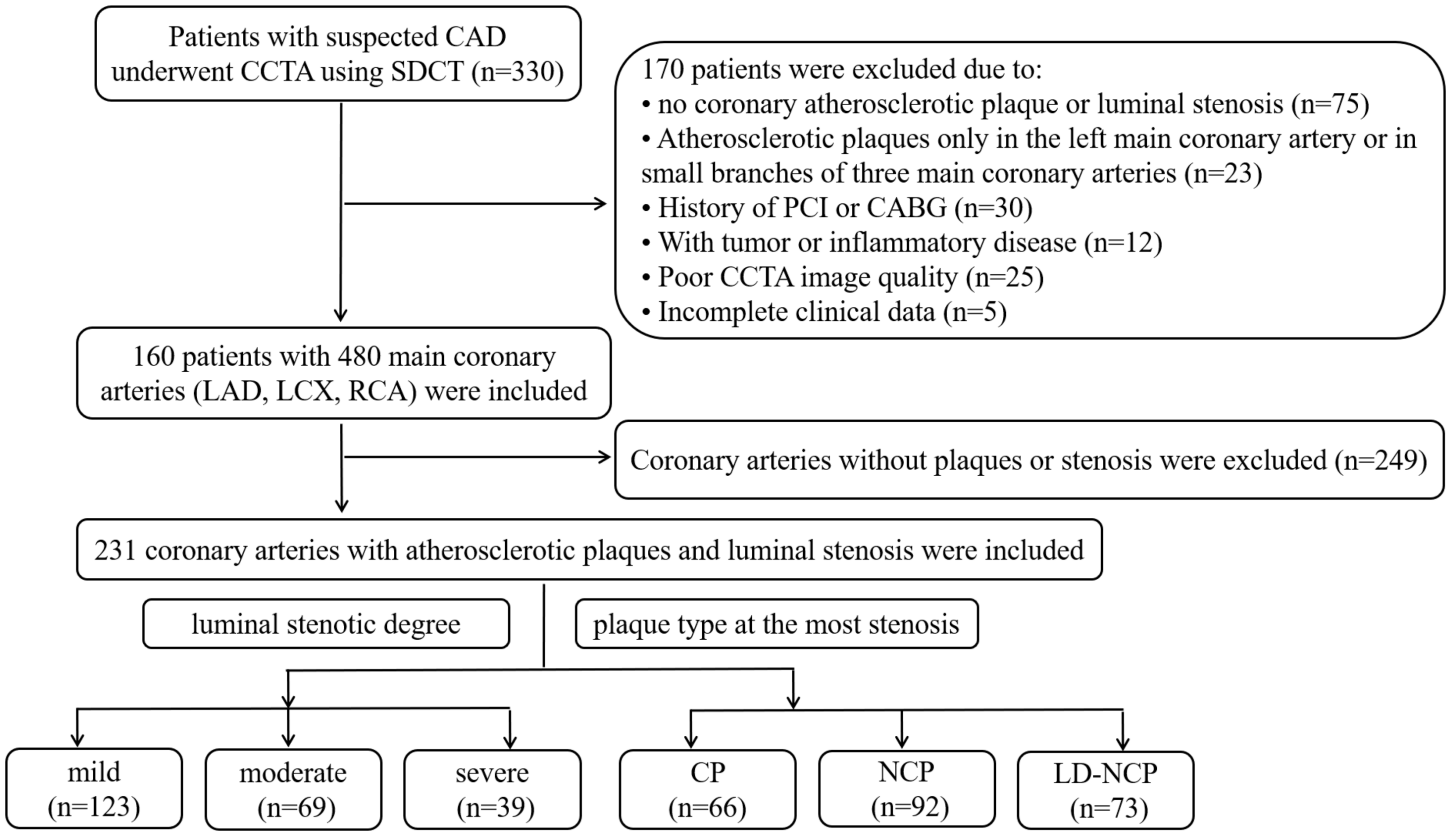
Study flowchart. CAD, coronary atherosclerotic heart disease; CCTA, coronary CT angiography; SDCT, dual-layer spectral detector computed tomography; PCI, percutaneous coronary intervention; CABG, coronary artery bypass graft; LAD, left anterior descending artery; LCX, left circumflex artery; RCA, right coronary artery; CP, calcified plaques; NCP, non-calcified plaque; and LD-NCP, low-density non-calcified plaque.

A total of 330 consecutive patients with suspected CAD underwent CCTA using dual-layer spectral detector CT (SDCT) at the First Affiliated Hospital of Harbin Medical University from April to November 2021. A total of 170 patients were excluded according to the following exclusion criteria: no coronary atherosclerotic plaque or luminal stenosis, or only the left main coronary artery or coronary branches of three main coronary arteries with atherosclerotic plaques; a history of percutaneous coronary intervention (PCI) or coronary artery bypass graft (CABG); malignant tumor or severe inflammatory disease; poor CCTA image quality; and incomplete clinical data. The remaining 160 patients had 480 main coronary arteries, including the LAD artery, LCX artery, and RCA. Among them, only 231 branches had atherosclerotic plaques and luminal stenosis. Therefore, the other 249 branches without plaques or stenosis were excluded. All CCTA images were independently evaluated for plaque types and luminal stenosis according to previous studies (13, 14) by two radiologists who had more than 10 years of experience in cardiac imaging diagnosis. The consensus was reached when there were different opinions. Finally, there were 123 coronary arteries with mild stenosis, 69 arteries with moderate stenosis, and 39 arteries with severe stenosis. In terms of plaque types, there were 66 coronary arteries with calcified plaques (CPs), 92 arteries with non-calcified plaque (NCP), and 73 arteries with low-density non-calcified plaque (LD-NCP). The study flow chart is shown in Figure 1. This retrospective study was approved by the ethics committee of the First Affiliated Hospital of Harbin Medical University (No. 202214).

### CCTA scan protocol and image reconstruction

The retrospective ECG-gated CCTA scans were performed on SDCT (IQon Spectral CT, Philips Healthcare, Best, The Netherlands). A bolus of 60–80 mL non-ionic contrast media (iobitridol; 350 mg iodine/ml, Guerbet, Roissy, France) was injected into the median cubital vein at a flow rate of 4–5 mL/s via a high-pressure injector with binocular tube (Ulrich, Germany), followed by 30 mL of saline flush. When the descending aorta reached the 110HU threshold, the CCTA scan was triggered automatically. The scan protocol was as follows: 120kVp tube voltage, automatic tube current modulation, 0.9 mm slice thickness and slice interval, 64 × 0.625 mm detector collimation, 270 ms tube rotation time, and 512 × 512 matrix. The original data were reconstructed, including both conventional images (CIs) and virtual monoenergetic images (VMIs) from 40 keV to 100 keV with a 10 keV interval. All data were transferred to the post-processing workstation (IntelliSpace Portal Vision 10, Philips Healthcare, Best, The Netherlands).

### PCAT attenuation parameter measurement

**Figure 2:**
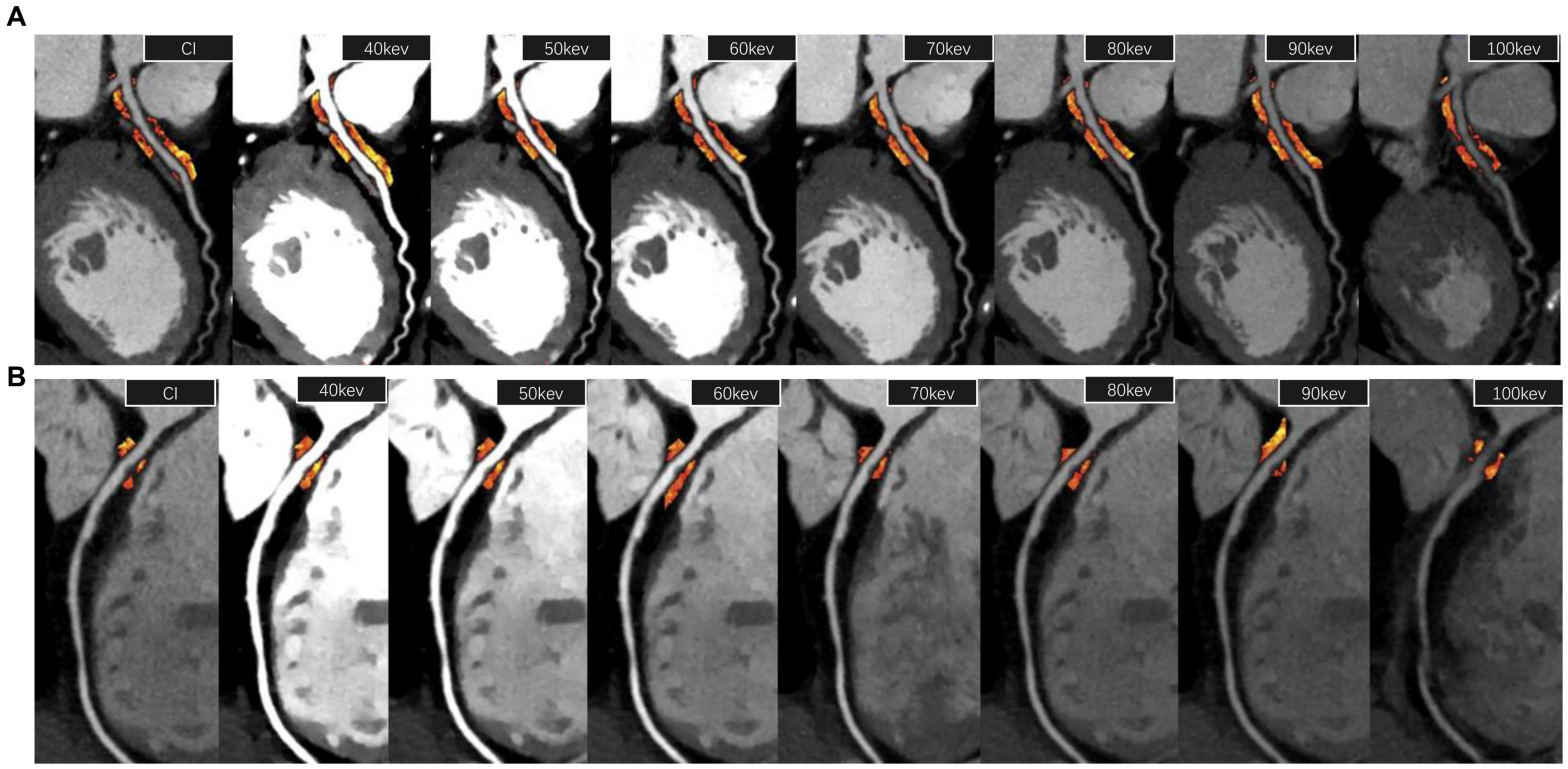
PCAT attenuation parameter measurement. **(A)** The measurement of proximal FAI of the left anterior descending artery (40 mm length from the bifurcation of the left main coronary artery) on a 120 kVp conventional image and 40–100 keV virtual monoenergetic images, respectively. **(B)** The measurement of peri-plaque FAI around a low-density non-calcified plaque at the most stenosis of the lumen of the right coronary artery. FAI, fat attenuation index.

Proximal and peri-plaque FAI of stenosis coronary arteries were measured both on CI and VMI by semi-automatic software (Shukun Technology, Beijing, China), which can automatically segment coronary arteries and identify PCAT. The measurement starting point, length, and width of proximal PCAT were set manually, completely consistent with the method used in our previous research (10). The starting point of the RCA measurement was set at 10 mm from the right coronary sinus ostium. LAD and LCX were measured from the bifurcation of the left main coronary artery. The measurement length was proximal 40 mm, and the measurement width was the mean diameter of the proximal coronary. Peri-plaque FAI was measured surrounding the plaque at the obvious stenosis. The measurement length was set according to the length of the plaque, and the measurement width was set according to the mean diameter of normal lumens at the proximal and distal ends of the plaque. Adipose tissue voxels on CI, 40 keV, 50 keV, 60 keV, 70 keV, 80 keV, 90 keV, and 100 keV images were identified with different thresholds, respectively: -190 ~ -30HU, −280 ~ -40HU, −260 ~ -40HU, −240 ~ -40HU, −220 ~ -30HU, −210 ~ -30HU, −200 ~ -30HU, and − 190 ~ -30HU. The slope of the spectral attenuation curve (λ) was computed: λ_40-70 KeV_ = (CT_40keV_-CT_70keV_)/30, λ_40-100 keV_ = (CT_40keV_-CT_100keV_)/60, and λ_70-100 KeV_ = (CT_70keV_-CT_100keV_)/30. The automatic PCAT attenuation parameter measurement is shown in Figure 2. In addition, peri-plaque FAI on CI, virtual non-contrast (VNC) image, and effective atomic number (Zeff) were also measured manually. The mean value of each parameter was obtained from three consecutive regions of interests (ROIs) with consistent size in the PCAT at the highest luminal stenosis.

### Statistical analysis

Continuous variables were expressed as the average ± standard deviation (SD). Normal distribution and homogeneity of variance tests on the data were performed. If the data were a normal distribution with homogeneity of variance, analysis of variance (ANOVA) was used for comparison among groups. Otherwise, a multi-variant ANOVA was performed. SPSS 26.0 (SPSS, Inc., Chicago, IL, USA) was used for statistical analysis. A *p*-value of <0.05 was considered statistically significant.

## Results

FAI gradually increased with the increase in monoenergetic level on VMI, whether it was proximal or peri-plaque FAI, whether it was mild, moderate, or severe lumen stenosis, whether it was CP, NCP, or LD-NCP (Supplementary Figure S1).

**Figure 3:**
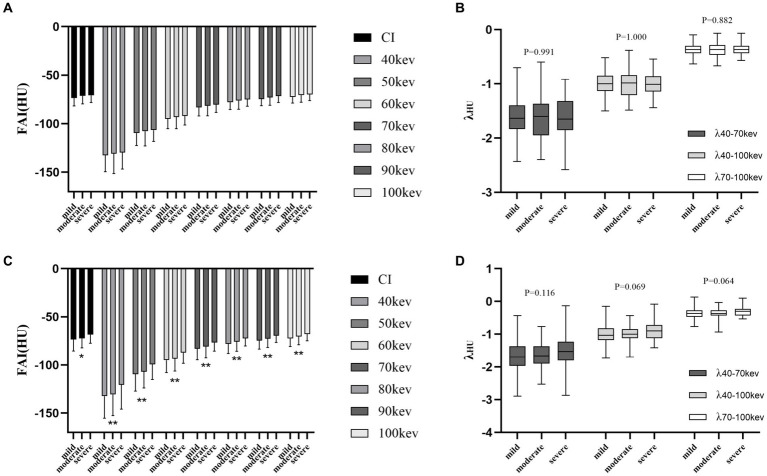
Differences in PCAT parameters at the luminal stenosis level. **(A)** Proximal FAI on CI and VMI. **(B)** Spectral attenuation slope of proximal PCAT at three monoenergetic intervals. **(C)** Peri-plaque FAI on CI and VMI. **(D)** Spectral attenuation slope of peri-plaque PCAT at three monoenergetic intervals. PCAT, pericoronary adipose tissue; FAI, fat attenuation index; CI, conventional image; and VMI, virtual monoenergetic image. *, *p* < 0.05; **, *p* < 0.01.

Among the different degrees of stenosis, both proximal and peri-plaque FAI increased with the stenosis degree of the coronary artery. Peri-plaque FAI_CI_ and FAI_VMI_ were significantly higher in severe stenosis than in mild and moderate stenosis (*p* < 0.05), while λ of proximal and peri-plaque PCAT attenuation was neither statistically different (*p* > 0.05) nor was the proximal FAI of vessels with different degrees of stenosis (*p* > 0.05). In severe stenosis, peri-plaque FAI was significantly higher than proximal FAI (*p* < 0.05), while they were not significantly different in mild and moderate stenosis (*p* > 0.05) (Figure 3) (Supplementary Tables S1, S2).

**Figure 4:**
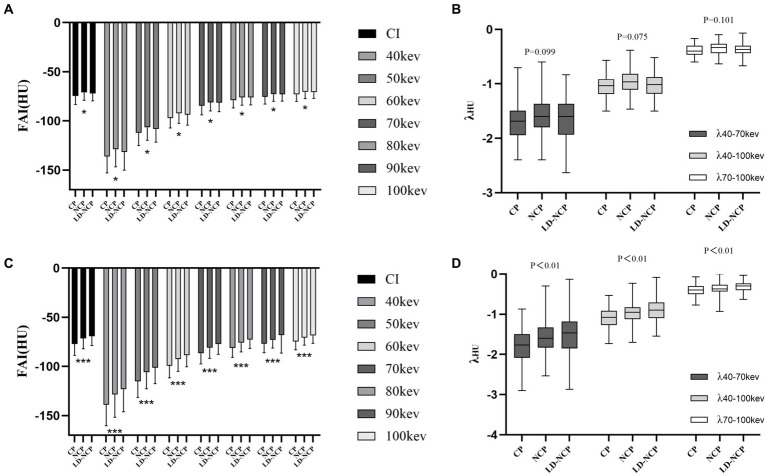
Differences in PCAT parameters at the plaque-type level. **(A)** Proximal FAI on CI and VMI. **(B)** Spectral attenuation slope of proximal PCAT at three monoenergetic intervals. **(C)** Peri-plaque FAI on CI and VMI. **(D)** Spectral attenuation slope of peri-plaque PCAT at three monoenergetic intervals. PCAT, pericoronary adipose tissue; FAI, fat attenuation index; CI, conventional image; VMI, virtual monoenergetic image; CP, calcified plaques; NCP, non-calcified plaque; LD-NCP, low-density non-calcified plaque. *, *p* < 0.05; ***, *p* < 0.001.

Among different plaque types, peri-plaque FAI_CI_, FAI_VMI_, and λ were significantly higher in LD-NCP and NCP than in CP (*p* < 0.01). The values were the highest in the LD-NCP group. Proximal FAI_CI_ and FAI_VMI_ were also significantly different in different plaque types (*p* < 0.05), but the highest FAI values were in the NCP group. λ of proximal PCAT attenuation was not statistically different among different plaque types (*p* > 0.05). In LD-NCP, peri-plaque FAI was significantly higher than the proximal FAI (*p* < 0.05), while they were not significantly different in CP and NCP (*p* > 0.05) (Figure 4) (Supplementary Tables S3, S4).

**Figure 5:**
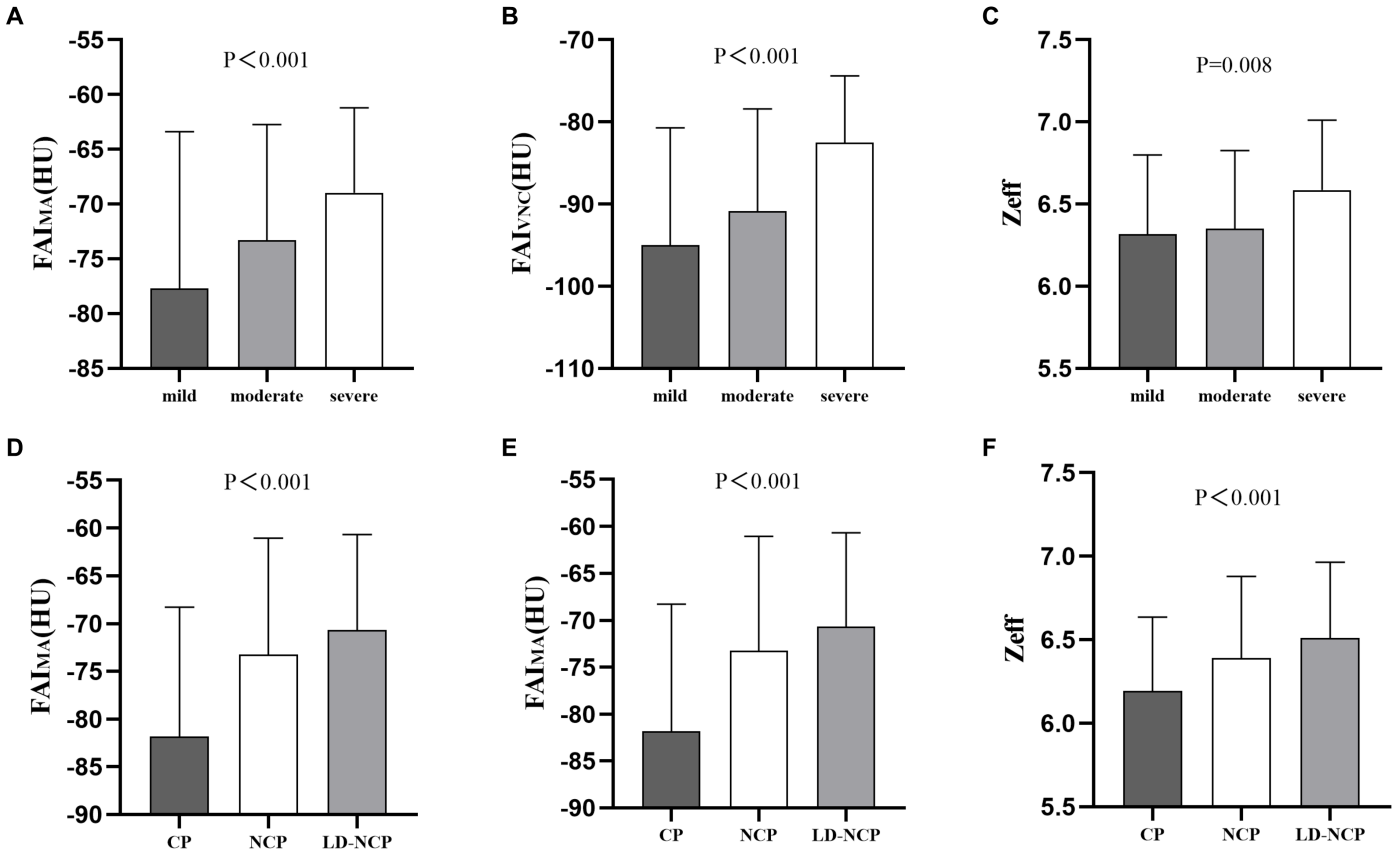
Differences in manually measured peri-plaque PCAT spectral parameters. **(A–C)** At luminal stenosis level. **(D–F)** At plaque-type level. FAI_MA_, fat attenuation index measured manually; FAI_VNC_, fat attenuation index on a virtual non-contrast image; Zeff, effective atomic number; CP, calcified plaques; NCP, non-calcified plaque; and LD-NCP, low-density non-calcified plaque.

The manually measured peri-plaque FAI on CI (FAI_MA_) was significantly positively correlated with the peri-plaque FAI_CI_ obtained automatically (*r* = 0.7265, *p* < 0.0001). Similar to the differences in automatically measured peri-plaque PCAT attenuation parameters between groups, the three manually measured peri-plaque PCAT spectral parameters also showed differences. The values of FAI_MA_, FAI_VNC_, and Zeff were as follows: severe stenosis > moderate stenosis > mild stenosis (*p* < 0.01) and LD-NCP > NCP > CP (*p* < 0.001) (Figure 5).

## Discussion

The present study explored the different roles between measured locations of PCAT attenuation in assessing coronary atherosclerosis. Peri-plaque PCAT attenuation was more valuable than proximal PCAT attenuation, and manual measurement of peri-plaque PCAT spectral quantitative parameters derived from SDCT also had important value. Therefore, peri-plaque measurement is recommended to obtain PCAT attenuation and spectral quantitative parameters, which are used to monitor and identify inflammation in patients with CAD. To the best of our knowledge, this is the first study to explore the measured location and measured method of PCAT quantitative parameters on SDCT.

CAD is caused by coronary atherosclerosis and luminal stenosis or occlusion. Due to the substantial risk of recurrent cardiovascular (CV) events, CAD remains the leading single cause of death worldwide (1, 15). The key driver of atherogenesis and atherosclerotic plaque rupture resulting in acute coronary syndrome (ACS) has been confirmed to be vascular inflammation (16). Therefore, the detection of coronary inflammation has important implications for CV risk stratification and targeted medical therapy, in order to improve patient prognosis. The routine non-invasive method for systemic inflammation monitoring is based on laboratory tests. It is convenient and sensitive, whereas it lacks specificity for coronary inflammation (17). Thus, it cannot be used for assessing the process of coronary atherosclerosis or the presence of vulnerable plaques. Several studies have proved that the CT attenuation index can represent the functional status of PCAT, which is closely related to coronary inflammation (3–5, 7). Hence, the study of evaluating coronary inflammation and related clinical issues through PCAT attenuation has become one of the research hotspots in recent years.

As part of perivascular adipose tissue (PVAT) and EAT, PCAT is also a functional sensor of coronary inflammation. PCAT is an active metabolic fat pool that is closely located around coronary arteries. It has a complex bidirectional paracrine pathway with a coronary artery wall. Dysfunctional PCAT secretes pro-inflammatory adipocytokines, causing vascular inflammation and leading to the formation of coronary atherosclerosis plaques. Vascular inflammation prevents lipid accumulation in PCAT by inhibiting preadipocyte differentiation (4, 18, 19). They are affected by each other’s inflammation and functional status. Therefore, it is possible to detect vascular inflammation non-invasively through PCAT attenuation using CCTA. In this study, we first confirmed the differences in PCAT attenuation among different degrees of luminal stenosis and among different coronary atherosclerotic plaque types, respectively. The result was particularly significant among plaque types. Therefore, the stages of plaque development may be an ideal target for better and earlier monitoring of atherosclerosis (20). In this study, we divided atherosclerotic plaques that caused the maximal degree of luminal stenosis into three types according to the attenuation characteristics on CCTA images. Among them, NCP and LD-NCP are the foundations of CV events and help to determine an individual’s CV risk (21). This study on the analysis of the relationship between plaque types and PCAT attenuation found that the degree of inflammation around NCP and LD-NCP was higher than that of the CP group. This finding indicates that the inflammatory state of PCAT and the coronary artery is closely related to the instability of atherosclerotic plaques, while CP is relatively stable and the degree of inflammation around it is relatively mild.

In addition, different measured locations of PCAT have different results, which is the main finding of this study. In the CRISP-CT study, PCAT around the proximal RCA was selected as a representative imaging marker of coronary inflammation for the analysis of PCAT in each participant (6). The main reason is that the adipose tissue around the proximal segment of RCA is relatively abundant, and it is easy to sketch, extract, and measure. However, this method may have limitations for the evaluation of some diffuse or focal lesions. It may not be sufficient for evaluating diffuse lesions, while for evaluating focal lesions, it may not be accurate enough because it covers normal adipose tissue. Therefore, some scholars have selected to adopt PCAT around the proximal segments of three major coronary arteries for attenuation measurement. However, they found that PCAT of LAD and LCX were not associated with event-free survival, although both PCAT of LAD and LCX and the occurrence of CV were correlated with RCT PCAT (6). In Alexios SA et al.’s study, they examined the relationships between FAI and radial measurement distance from the vascular wall in patients with coronary atherosclerosis compared to healthy individuals. The results showed that there was no difference in FAI 2 cm away from the coronary wall between the two groups. However, they found that FAI around the culprit lesion was higher than FAI proximal to the lesion. Therefore, they proposed that there is a gradient in adipocyte size and the expression of adipose genes moving from PVAT adjacent to coronary arteries to adipose tissue further away from the coronary wall, which makes a parallel shift of FAI to more negative values moving away from the coronary wall (4). Another study also found different FAI values with the radial distance for PCAT proximal to the coronary artery. As shown, FAI_ref_ (within the radial distance from the outer vessel wall equal to the coronary vessel diameter) proximal to LAD could better represent the inflammation of culprit lesions in patients with ACS than FAI with any other radial distances proximal to the coronary arteries or FAI around culprit lesions (22). In our study, we confirmed that there are indeed differences in the PCAT attenuation at different locations. What is different is that we have confirmed that the change in PCAT attenuation around the plaque is more significant than that of proximal coronary arteries, especially in severe stenostic vessels and LD-NCP. A possible explanation for this result may be that the lesion is mainly on a single coronary artery in most participants in this study. The paracrine signal sent to PCAT from the vascular inflammation is easier to be limited around the plaque and then transmitted to PCAT around the inflamed coronary arteries and other locations. Therefore, peri-plaque PCAT attenuation is a more robust and easily accessible measurement of coronary inflammation. It has a higher diagnostic value in CAD patients as compared to proximal PCAT attenuation.

Furthermore, SDCT was used, and several spectral parameters of PCAT were obtained to assess coronary arteriosclerosis in this study, which is another innovation. In our previous study (10), we confirmed that PCAT attenuation parameters obtained using dual-layer SDCT can aid in distinguishing patients with and without CAD, which might predict the formation of atherosclerotic plaques before they appear. Most previous studies on PCAT attenuation in evaluating coronary atherosclerosis and CV events were performed by conventional polyenergetic CT. In this study, PCAT attenuation parameters from CI and VMI were both adopted. In addition, manually measured peri-plaque PCAT attenuation on CI and VNC images and Zeff of peri-plaque PCAT were also used for assessing coronary atherosclerosis. The results confirmed that peri-plaque PCAT spectral parameters measured manually have significant value in estimating coronary atherosclerosis, which is similar to the results of automatically measured peri-plaque PCAT attenuation. As the most commonly used imaging detection method, conventional CCTA can offer information on the morphological manifestation of coronary plaques and the structural change in vascular walls. However, SDCT can provide more functional information and quantitative parameters. It achieves accurate “homologous, simultaneous, codirectional and synchronous” spectral scanning based on collecting low- and high-energy data simultaneously using a dual-layer detector (23, 24). Therefore, it can derive more real and accurate data for measurement and diagnosis based on non-invasive multimodal images. Besides, it can also supply more spectral quantitative parameters. In this study, the combination of multiple PCAT spectral parameters can better diagnose and assess the severity of luminal stenosis and atherosclerotic plaques.

This study has some limitations. First, this is a single-center retrospective study, which is based on vessel level and the specific device. Therefore, culprit plaques, follow-up prognosis, and the influence of different types and brands of CT imaging devices were not analyzed, which requires further and prospective research in the future. Second, the laboratory test was not included in this study. The calcified score and invasive coronary angiography were not adopted, either. Thus, the level of coronary inflammation and the severity of coronary atherosclerosis could not be assessed comprehensively and accurately. Third, only the relationship between the longitudinal location of PCAT parameters and coronary atherosclerosis was analyzed, while PCAT attenuation with different radial distances was not measured. Although peri-plaque PCAT attenuation parameters are expected to be used as a standard biomarker for evaluating the severity of coronary atherosclerosis, further randomized controlled trials with a large sample and detailed analysis are required to verify this hypothesis.

In conclusion, peri-plaque PCAT is more valuable than proximal PCAT in assessing coronary atherosclerosis. Spectral parameters of peri-plaque PCAT can further improve the diagnostic accuracy of coronary atherosclerosis. The quantitative parameters related to peri-plaque PCAT attenuation are expected to become standard biomarkers and predictors for assessing plaque vulnerability and the hemodynamic characteristics of coronary atherosclerosis.

## Data availability statement

The original contributions presented in the study are included in the article/Supplementary material, further inquiries can be directed to the corresponding author.

## Ethics statement

The studies involving human participants were reviewed and approved by the First Affiliated Hospital of Harbin Medical University (No. 202214). Written informed consent was not required as per local legislation and institutional requirements.

## Author contributions

YJ: Conceptualization, Methodology, Writing – original draft, Writing – review & editing, Data curation, Formal analysis. LZ: Data curation, Methodology, Writing – original draft, Formal analysis. MX: Data curation, Formal analysis. XZ: Data curation, Formal analysis, Writing – original draft. XX: Conceptualization, Project administration, Writing – original draft, Writing – review & editing.
