# Supplementary figures and images for: Evaluation of peri-plaque pericoronary adipose tissue attenuation in coronary atherosclerosis using a dual-layer spectral detector CT
Source: Front Med (Lausanne). 2024 Mar 11;11:1357981. doi: 10.3389/fmed.2024.1357981 (PMC10964482; doi:10.3389/fmed.2024.1357981)

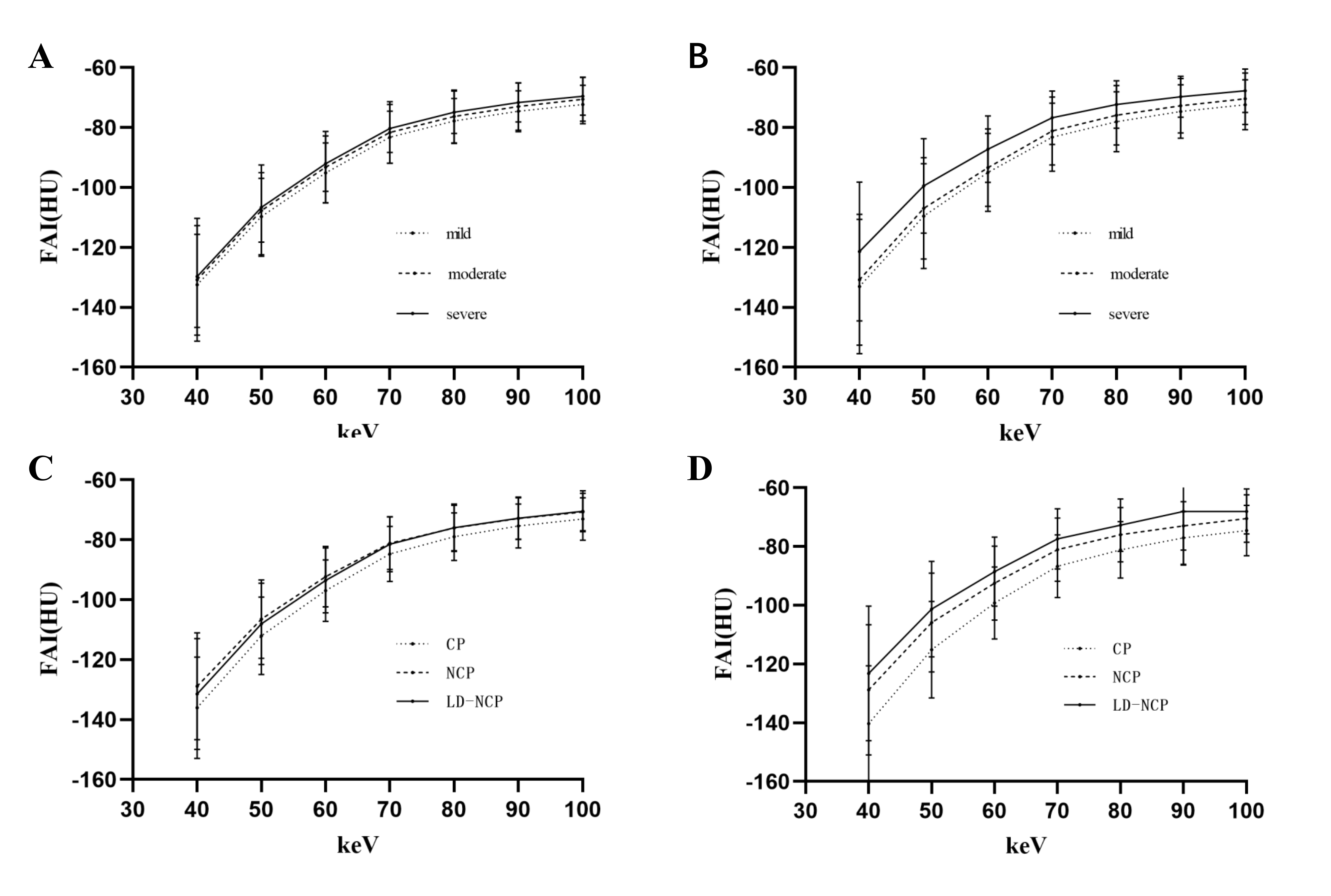

Supplement: Supplementary file 1 [file Image_1.TIF]
