# Supplementary material for: Evaluation of peri-plaque pericoronary adipose tissue attenuation in coronary atherosclerosis using a dual-layer spectral detector CT
Source: Front Med (Lausanne). 2024 Mar 11;11:1357981. doi: 10.3389/fmed.2024.1357981 (PMC10964482; doi:10.3389/fmed.2024.1357981)
Supplement: Supplementary file 2 [file Table_1.DOCX]

**Supplemental Tables**

**Table S1** Proximal PCAT attenuation parameters among luminal stenostic degrees

| PCAT attenuation parameter | mild  (n=123) | moderate  (n=69) | severe  (n=39) | *F* | *P* value |
| --- | --- | --- | --- | --- | --- |
| FAI_CI_ (HU) | -73.61±8.02 | -71.30±8.24 | -70.76±8.05 | 2.886 | 0.058 |
| FAI_VMI_ |  |  |  |  |  |
| FAI_40keV_ (HU) | -132.49±16.90 | -130.76±20.53 | -129.20±16.98 | 0.436 | 0.647 |
| FAI_50keV_ (HU) | -109.71±12.78 | -107.66±15.28 | -106.61±11.56 | 1.022 | 0.362 |
| FAI_60keV_ (HU) | -95.11±10.01 | -93.18±11.93 | -92.07±9.31 | 1.547 | 0.215 |
| FAI_70keV_ (HU) | -83.24±8.72 | -81.60±10.30 | -80.25±8.01 | 1.835 | 0.162 |
| FAI_80keV_ (HU) | -77.81±7.48 | -76.27±8.85 | -74.89±7.11 | 2.311 | 0.101 |
| FAI_90keV_ (HU) | -74.52±6.84 | -72.95±7.95 | -71.61±6.47 | 2.805 | 0.063 |
| FAI_100keV_ (HU) | -72.29±6.43 | -70.53±7.33 | -69.53±6.30 | 3.151 | 0.055 |
| λ |  |  |  |  |  |
| λ_40-70keV_ | -1.64±0.34 | -1.63±0.39 | -1.64±0.35 | 0.009 | 0.991 |
| λ_40-100keV_ | -1.00±0.21 | -1.00±0.24 | -1.00±0.20 | 0.001 | 1.000 |
| λ_70-100keV_ | -0.36±0.11 | -0.36±0.12 | -0.35±0.10 | 0.126 | 0.882 |

PCAT, pericoronary adipose tissue; FAI, fat attenuation index; CI, conventional image; VMI, virtual monoenergentic image; λ, the slope of spectral attenuation curve.

**Table S2** Peri-plaque PCAT attenuation parameters among luminal stenostic degrees

| PCAT attenuation parameter | mild  (n=123) | moderate  (n=69) | severe  (n=39) | *F* | *P* value |
| --- | --- | --- | --- | --- | --- |
| FAI_CI_ (HU) | -73.61±11.84 | -72.49±9.71 | -68.56±8.91 | 3.239 | 0.041 |
| FAI_VMI_ |  |  |  |  |  |
| FAI_40keV_ (HU) | -133.03±22.40 | -130.75±21.89 | -121.36±23.16 | 4.042 | 0.009 |
| FAI_50keV_ (HU) | -109.57±17.56 | -106.97±16.96 | -99.43±15.73 | 5.213 | 0.006 |
| FAI_60keV_ (HU) | -94.92±13.01 | -93.39±12.91 | -87.23±11.08 | 5.466 | 0.005 |
| FAI_70keV_ (HU) | -83.21±11.31 | -81.15±11.40 | -76.69±8.92 | 5.263 | 0.006 |
| FAI_80keV_ (HU) | -78.04±9.98 | -75.88±9.89 | -72.28±7.90 | 5.454 | 0.005 |
| FAI_90keV_ (HU) | -74.61±8.94 | -72.72±9.11 | -69.66±6.89 | 4.963 | 0.008 |
| FAI_100keV_ (HU) | -72.34±8.35 | -70.34±8.58 | -67.74±7.25 | 4.894 | 0.008 |
| λ |  |  |  |  |  |
| λ_40-70keV_ | -1.66±0.44 | -1.65±0.43 | -1.48±0.55 | 2.174 | 0.116 |
| λ_40-100keV_ | -1.01±0.27 | -1.00±0.27 | -0.89±0.30 | 2.706 | 0.069 |
| λ_70-100keV_ | -0.36±0.14 | -0.36±0.16 | -0.29±0.14 | 2.779 | 0.064 |

PCAT, pericoronary adipose tissue; FAI, fat attenuation index; CI, conventional image; VMI, virtual monoenergentic image; λ, the slope of spectral attenuation curve.

**Table S3** Proximal PCAT attenuation parameters among different plaque types

| PCAT attenuation parameter | CP  (n=66) | NCP  (n=92) | LD-NCP  (n=73) | *F* | *P* value |
| --- | --- | --- | --- | --- | --- |
| FAI_CI_ (HU) | -74.77±8.39 | -71.04±7.92 | -72.09±7.48 | 4.372 | 0.014 |
| FAI_VMI_ |  |  |  |  |  |
| FAI_40keV_ (HU) | -136.06±16.93 | -128.87±17.81 | -131.49±18.56 | 3.155 | 0.044 |
| FAI_50keV_ (HU) | -112.07±12.93 | -106.48±13.11 | -108.02±13.61 | 3.537 | 0.031 |
| FAI_60keV_ (HU) | -96.95±10.21 | -92.33±10.11 | -93.52±10.86 | 3.945 | 0.021 |
| FAI_70keV_ (HU) | -84.71±9.17 | -81.13±8.80 | -81.43±9.18 | 3.449 | 0.033 |
| FAI_80keV_ (HU) | -78.93±7.89 | -75.98±7.93 | -76.07±7.65 | 3.047 | 0.040 |
| FAI_90keV_ (HU) | -75.39±7.37 | -72.80±7.14 | -72.86±6.90 | 3.263 | 0.049 |
| FAI_100keV_ (HU) | -71.07±7.08 | -70.46±6.80 | -70.70±6.26 | 3.266 | 0.040 |
| λ |  |  |  |  |  |
| λ_40-70keV_ | -1.71±0.33 | -1.59±0.35 | -1.66±0.38 | 2.337 | 0.099 |
| λ_40-100keV_ | -1.04±0.20 | -0.96±0.22 | -1.01±0.23 | 2.618 | 0.075 |
| λ_70-100keV_ | -0.38±0.11 | -0.34±0.11 | -0.36±0.12 | 2.314 | 0.101 |

PCAT, pericoronary adipose tissue; FAI, fat attenuation index; CI, conventional image; VMI, virtual monoenergentic image; CP, calcified plaques; NCP, non-calcified plaque; LD-NCP, low-density non-calcified plaque.

**Table S4** Peri-plaque PCAT attenuation parameters among different plaque types

| PCAT attenuation parameter | CP  (n=66) | NCP  (n=92) | LD-NCP  (n=73) | *F* | *P* value |
| --- | --- | --- | --- | --- | --- |
| FAI_CI_ (HU) | -77.16±11.53 | -71.49±10.43 | -69.23±9.40 | 10.55 | ＜0.001 |
| FAI_VMI_ |  |  |  |  |  |
| FAI_40keV_ (HU) | -140.30±19.66 | -128.78±22.21 | -123.24±22.92 | 11.040 | ＜0.001 |
| FAI_50keV_ (HU) | -115.12±16.41 | -105.87±16.87 | -101.26±16.30 | 12.523 | ＜0.001 |
| FAI_60keV_ (HU) | -99.21±12.28 | -92.43±12.63 | -88.56±11.76 | 13.324 | ＜0.001 |
| FAI_70keV_ (HU) | -86.69±10.68 | -81.02±10.78 | -77.34±10.27 | 13.645 | ＜0.001 |
| FAI_80keV_ (HU) | -81.12±9.60 | -75.96±9.29 | -72.72±8.97 | 14.346 | ＜0.001 |
| FAI_90keV_ (HU) | -77.06±9.05 | -72.96±8.29 | -68.08±18.22 | 9.060 | ＜0.001 |
| FAI_100keV_ (HU) | -74.54±8.57 | -70.47±8.06 | -68.02±7.69 | 11.399 | ＜0.001 |
| λ |  |  |  |  |  |
| λ_40-70keV_ | -1.78±0.40 | -1.59±0.44 | -1.53±0.51 | 5.985 | 0.003 |
| λ_40-100keV_ | -1.09±0.25 | -0.97±0.27 | -0.92±0.30 | 7.259 | 0.001 |
| λ_70-100keV_ | -0.40±0.13 | -0.35±0.15 | -0.31±0.13 | 7.566 | 0.001 |

PCAT, pericoronary adipose tissue; FAI, fat attenuation index; CI, conventional image; VMI, virtual monoenergentic image; CP, calcified plaques; NCP, non-calcified plaque; LD-NCP, low-density non-calcified plaque.
